# Editorial: Unveiling distinctions: active tuberculosis versus latent tuberculosis infection - immunological insights, biomarkers, and innovative approaches

**DOI:** 10.3389/fcimb.2025.1659311

**Published:** 2025-08-22

**Authors:** Ying Luo

**Affiliations:** Department of Immunology, University of Texas Southwestern Medical Center, Dallas, TX, United States

**Keywords:** active tuberculosis, latent tuberculosis infection, diagnosis, immunological insights, biomarkers

Tuberculosis (TB) remains one of the leading causes of mortality worldwide, with an estimated 1.25 million deaths and 10.8 million cases in 2023 (Organization, 2024). The ability to accurately distinguish active TB (ATB) from latent TB infection (LTBI) is critical for the effective management and control of the disease. Rapid and precise diagnosis of TB not only facilitates timely treatment but also plays a pivotal role in interrupting the transmission of *Mycobacterium tuberculosis* (MTB). However, the development of sensitive and reliable diagnostic approaches for TB remains a significant unmet need, underscoring the necessity for innovative methods that can be translated into clinical practice.

This Research Topic aims to attract innovative studies that contribute to the development of a comprehensive diagnostic toolkit enabling clinicians to distinguish ATB from LTBI with heightened precision. In this Research Topic, a total of 11 papers, including 9 original research articles and 2 reviews, were published. Pan et al. reported that false-negative MPT64 antigen results in MTB cultures can be attributed to mutations within the mpt64 gene. Gong et al., using single-cell RNA sequencing and TCR profiling, revealed that lymphopenia, T -cell exhaustion, and TCR repertoire features contribute to the pathogenesis of hematogenous disseminated TB. Liu et al. demonstrated that lower systemic immune inflammation, assessed through NHANES and transcriptomic datasets, correlates with LTBI. Another study by Liu et al. developed a diagnostic model for tuberculous meningitis using laboratory parameters, achieving an area under the curve of 0.86. Ren et al. established a diagnostic model based on four autophagy-related genes that effectively distinguishes ATB from other conditions. García et al. underscored the importance of contact tracing and follow-up of household children, particularly when the index case is smear-positive. Mensah et al. developed a diagnostic model integrating eight serum cytokine/chemokine biomarkers to distinguish ATB from LTBI in a Ghanaian cohort. Ou et al. evaluated and confirmed that the TB Pro assay offers a sensitive and specific approach for simultaneous mycobacterial identification and comprehensive drug-resistance profiling, demonstrating robust performance on both cultured isolates and direct clinical specimens. Zhang et al. utilized transcriptomic and proteomic profiling of host NK cells to delineate distinct immune states across TB infection statuses. Additionally, two review articles summarized recent advances in TB diagnostics and management. Yang et al. highlighted progress in blood-based biomarkers and emerging diagnostic technologies for ATB, while Gunasekaran et al. reviewed the utility of inflammatory biomarkers in the detection and management of LTBI.

Collectively, these studies underscore the critical roles of host factors, pathogen characteristics, and their interplay in shaping the transition between latent and active TB, thereby advancing diagnostics and deepening our understanding of TB immunopathogenesis. Moreover, these observations highlight the growing importance of incorporating immune profiling and artificial intelligence into TB diagnostics, as demonstrated by a series of recent reports in this field (Luo et al., 2020; Luo et al., 2022; Li et al., 2023; Luo et al., 2023; Wang et al., 2024; Wang et al., 2025). However, significant limitations in TB diagnostics persist. First, rigorous validation of candidate biomarkers in diverse, well-powered cohorts is essential to facilitate the translation of promising markers from individual laboratories to standardized and commercially viable clinical assays. This underscores the need to establish stringent criteria for selecting candidates with true diagnostic utility. Second, there is a need to facilitate investigations into the immunopathology of TB to enable the dissection of molecular dynamics throughout the disease course. Such mechanistic insights will not only advance our understanding of TB pathogenesis but also inform the development of clinically actionable biomarkers and therapeutic targets. Moreover, improving the quality and reproducibility of clinical testing is imperative to reducing variability arising from uncontrolled data quality, which often complicates interpretation. Researchers should devote attention to investigating the biological underpinnings of outlier data rather than dismissing them as noise. The heterogeneity observed in TB underscores the importance of focusing on individual-level variations rather than solely on cohort-level statistical significance. Rare findings in clinical data may, in fact, reflect meaningful biological phenomena requiring further exploration. Detailed analyses at the individual patient level, alongside cohort-based assessments, will ultimately yield insights that benefit both the broader TB population and individual patients. Finally, it is critical that researchers bridge observations from clinical settings with mechanistic insights from experimental models, fostering a translational pipeline that connects clinical phenomena to biological mechanisms. Such integrative efforts will be pivotal for advancing TB diagnostics and therapeutics in a manner that truly impacts patient care.
